# Comparing catch efficiency of five models of pot for use in a Newfoundland and Labrador cod fishery

**DOI:** 10.1371/journal.pone.0199702

**Published:** 2018-06-27

**Authors:** Phillip Meintzer, Philip Walsh, Brett Favaro

**Affiliations:** 1 Department of Ocean Sciences, Memorial University of Newfoundland, St. John’s, Newfoundland and Labrador, Canada; 2 Fisheries and Marine Institute of Memorial University of Newfoundland, St. John’s, Newfoundland and Labrador, Canada; Swedish University of Agricultural Science, SWEDEN

## Abstract

Sustainability of commercial fisheries is best achieved when fishing gears are selective and have low impacts on bottom habitat. Pots (baited traps) are a fishing technology that typically has lower impacts than many other industrial gears. In this study we compared the efficiency of five models of pots (baited traps) designed to catch Atlantic cod (*Gadus morhua*) for use in Newfoundland and Labrador (NL)’s expanding cod fishery. We compared catch per unit effort (CPUE) and total lengths of cod across each pot type, as well as bycatch rates of each model. All pot types were successful at catching cod, but two models (the modified Newfoundland pot, and a four-entrance pot of our design) had highest CPUE. Specifically, we found that modifying Newfoundland pots increased their CPUE by 145% without a corresponding increase in bycatch. None of the pot types produced substantial amounts of bycatch. This study demonstrated that potting gear is an effective way to catch cod in NL, and that there is flexibility in which pot fishers can use, depending on the layout of their fishing vessel.

## Introduction

Pots (also commonly referred to as traps), are cage-like, stationary fishing gears widely used in commercial fisheries throughout the world [1–4]. Pots are transportable, and typically use bait to attract target species, with retention devices to prevent their escape [5]. The benefits of using pots include decreased rates of bycatch [6], minimal impacts to marine habitats, and a reduced contribution to ghost fishing (when constructed with biodegradable twine) when compared to gillnets [5]. Pots have also been classified as a ‘Low Impact and Fuel Efficient’ (LIFE) fishing gear, because they require less fuel to harvest than towed fishing gears such as trawls and dredges [5]. In addition, fish trapped in pots remain alive and unensnared until the gear is retrieved [7,8]. As a result, meat quality of pot-caught fish often exceeds that of other gears where the act of capture imposes immediate damage to the fish [6]. In addition, trapped species not intentionally targeted by fishing (i.e. bycatch) can generally be returned to the water with a high chance of survival [5].

In Canada, pots are currently used to capture many species, including spot prawns (*Pandalus platyceros*) [9,10] and sablefish (*Anoplopoma fimbria*) [11] in British Columbia, and snow crabs (*Chionoecetes opilio)* in Newfoundland and Labrador (NL) [12]. These fisheries are widely regarded as highly sustainable, and they have been recognized by eco-certifications such as Oceanwise and Seachoice [13]. In the case of spot prawns and sablefish in particular, collectively these fisheries produced a landed value of 50.6 million dollars in 2013, and both species fetch a high market value per kilogram [14]. These fisheries demonstrate the viability of pots as a foundational fishing technology for sustainable fisheries.

In NL, there have been calls to establish a re-emerging fishery for Atlantic cod around the concept of value-maximization—using gears and fishing techniques that maximize quality and enable fishers to achieve higher landed value for their catch [15]. Large-scale commercial fishing for Atlantic cod in NL ceased with the moratorium on the cod fishery in 1992, but recent increases in the population [16] have resulted in fishers and some members of the general public to call for increases in quota and corresponding increases in fishing effort [17,18]. If an expanding fishery is to be built on high-value catch, the industry will need fishing gears capable of ensuring high quality of captured fish with sufficient efficiency to be economically viable.

Pots represent a reduced-impact gear that could play an increased role in an expanded NL cod fishery. While pots are not widely used in NL to catch cod, where only a small group of commercial fishers on Fogo Island, NL, have been using experimental pots since 2007 [19] as part of the annual stewardship fishery for cod (i.e. the small commercial fishery that has permission to occur every year despite the ongoing moratorium) [20,21]. However, potting has not yet been widely adopted as the primary fishing gear in the region, with the majority of fishers still using gillnets or hand-lines.

For pots to be a viable fishing gear, they must be designed around efficiency, selectivity, usability and safety, and ease of procurement. Each of these factors ultimately affect profitability and environmental impact of the gear, and therefore, the likelihood that fishers will adopt it. Modifications to any part of a pot can drastically alter its catch composition [22]. For example, pots with smaller mesh size were found to have greater catch rates than pots with larger mesh in an Australian fishery [23]. In Norway, adding floats to pots for Atlantic cod reduced the bycatch of crustaceans when compared to bottom set pots [24]. Modifying the ability of organisms to exit the pot is important too—for example, escape mechanisms have been found to reduce the catch of undersized snow crab (*Chionoecetes opilio*) in Canada [12], and the use of funnel shaped entrances resulted in an increased catch of Atlantic cod compared to entrances lacking funnels, by preventing escapes from pots fished in the Baltic Sea [22]. Even pot orientation matters—in a previous study, we found that existing pots needed to ensure at least one entrance is in-line with the downstream current direction to increase successful entries by Atlantic cod [7]. These examples demonstrate the considerable effect that pot designs can have on their catch efficiency and composition.

In this study we assessed the effectiveness of five different types of pots at catching Atlantic cod, using catch and length data collected during field trials of experimental pots aboard commercial fishing vessels, over the course of two consecutive fishing seasons during the summers of 2015 and 2016. We tested these gears in real-world field conditions—aboard industry vessels fishing during the annual stewardship fishery [21]. We compared catch-per-unit-effort (CPUE) and body sizes of captured cod across pot types. In addition, we compared bycatch rates across pot types, and qualitatively assessed their ease of use at sea. Finally, we obtained and reported summary statistics of opportunistically-acquired data comparing landed fish quality across pots, gillnets, and hook-and-line gears.

## Materials & methods

### Field studies

**Fig 1:**
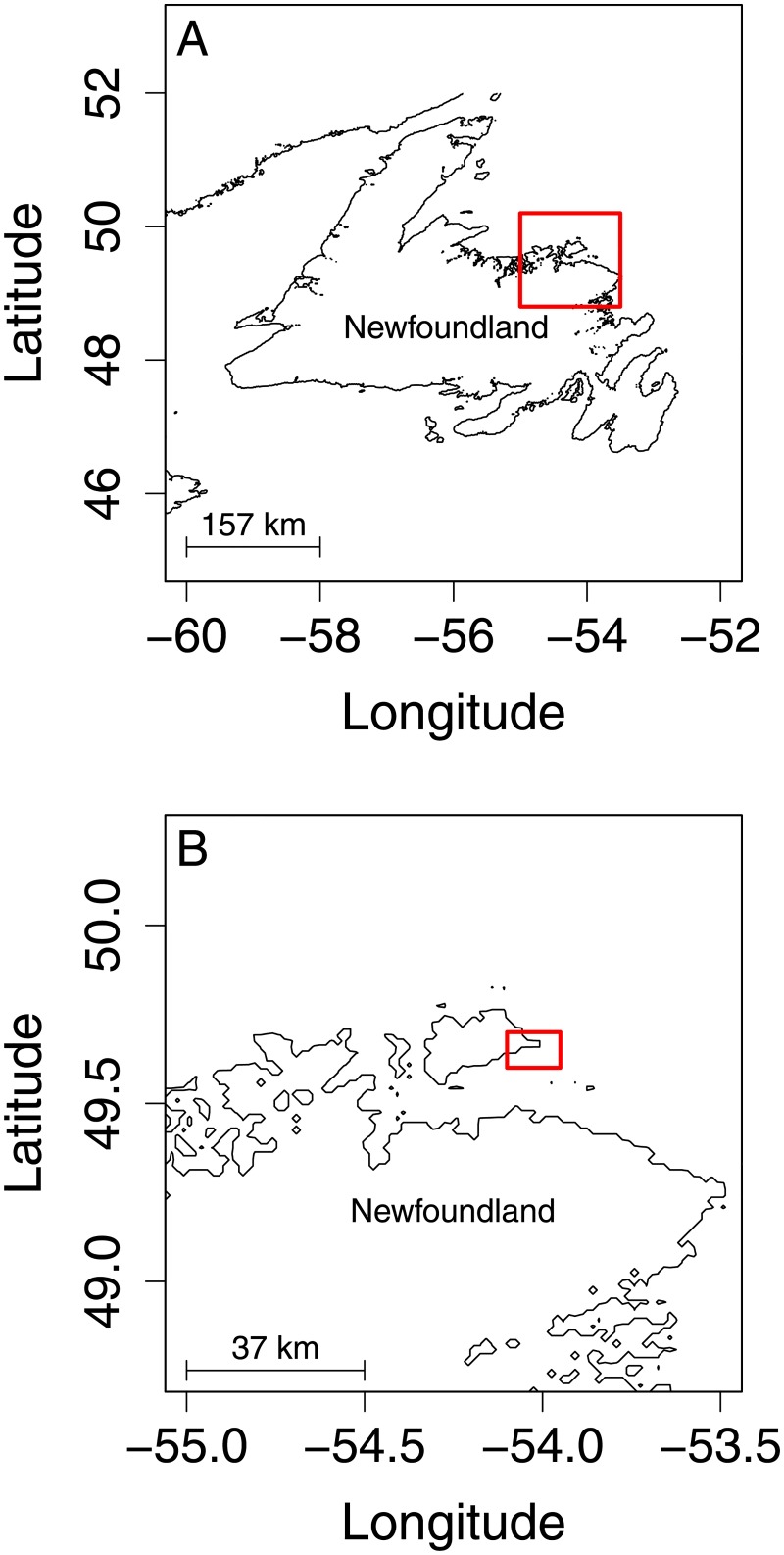
Map of our research site, off of the southern coast of Fogo Island, NL, for both field studies in 2015 and 2016. The red rectangle on the top map (A) indicates the location of Fogo Island relative to Newfoundland, and the red rectangle on the bottom map (B) encompasses the greater fishing area where we deployed our cod pots during both field studies.

We conducted two separate field experiments comparing the CPUE of Atlantic cod across several pot designs. Both studies took place within 5 km of southern Fogo Island, NL (Fig 1). The first experiment occurred between Aug 20 and Sept 1, 2015, and the second between Aug 22 and Sept 2, 2016. We selected these dates so our experiment would take place during the annual stewardship fishery [20,25]. This enabled us to conduct our experiment aboard industry vessels conducting actual commercial fishing operations, meaning our CPUEs are likely to reflect realistic in-season fishing performance. Our experiments took place aboard the 10.4 m (34 foot) fishing vessels *Dean & Michael*, and the *Beverly Crystal*, operated by commercial cod fishers based in Seldom, NL. The project was reviewed and approved by Memorial University’s Institutional Animal Care Committee (Project # 15-03-BF). Permission to conduct this field study was granted under the fishing license conditions of our industry partner, as our study took place during the annual Atlantic cod stewardship fishery.

### Pot selection and development

In the first experiment we tested two models of pot: Newfoundland-style pots (hereafter NL), and Norwegian-style pots (hereafter NOR). We selected these pot types for comparison because both are currently in use in fisheries targeting cod; the former, by a small group of fishers on Fogo Island NL [26], and the latter by fishers in Norway and Sweden [27–30]. In our second experiment, we assessed five pot types: NL, NOR, modified NL (NL-mod), modified NOR (NOR-mod), and a four-entrance pot of our design (4-ent).

**Fig 2:**
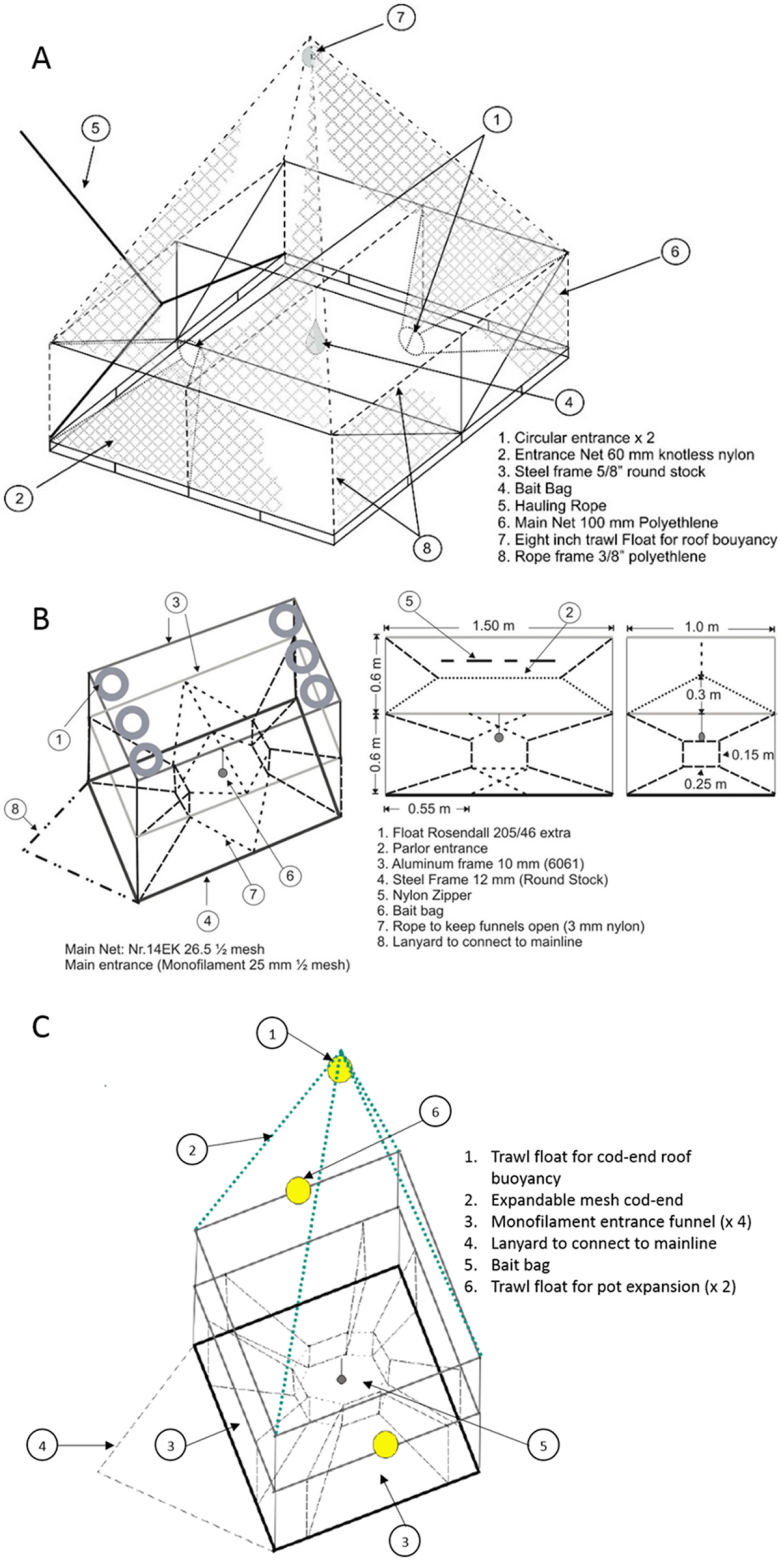
Diagrams of the different pot types used during our 2015, and 2016 field studies. Fig 2A pictures a Newfoundland (NL) pot. Fig 2B pictures a Norwegian (NOR) pot. Fig 2C represents a 4-ent pot. All figures represent pots as they would appear deployed on the sea bottom.

The NL pot was a large pot (2 x 2 x 1 m), with a heavy frame constructed of round reinforcing steel (Fig 2A). The frame of the NL pot was composed of a square bottom, connected to four collapsible steel beams which extend from a central pivot point to form the sides of the pot. The collapsibility allowed easy transportation and storage of the pot when not in use. The NL pot had two offset entrance funnels, typically constructed with 58 mm white diamond knotless nylon mesh. These funnels contained a metal retention device known as a trigger, which used long metal finger-like projections to allow one-way movement into the pot, and to prevent escape. The NL pots had a single bait bag suspended at the center of the pot, and contained a large expandable mesh roof, known as a cod-end, which extended upward during deployment using a flotation device. This netting panel (100 mm diamond polyethylene) covered the entire exterior of the pot and the netting was hung on the frame of the pot at approximately 65% of stretched mesh opening.

The NL-mod pot shared the same basic design as the NL pot, but with several modifications. We replaced the standard white 58 mm nylon mesh entrance funnels with clear 58 mm nylon diamond monofilament netting entrance funnels and removed the metal retention triggers. A mesh separator panel was added at the midway point up the vertical length of the pot using 58 mm mesh size black polyester netting, to divide the pot into upper and lower chambers. A slit in the dividing mesh allowed cod to enter the upper chamber. Finally, instead of a single bait bag suspended in the center of the pot, we used two smaller bait bags, each positioned in front of an entrance funnel.

The NOR pot was a two-chambered pot consisting of three rectangular frames in a collapsible structure (Fig 2B, see also [7]). The bottom frame was made of steel (to provide weight on the bottom of the pot), and the two frames above were both made of aluminum. Floatation attached to the top of the frame caused the pot to expand vertically underwater when deployed. The exterior netting on the pot was constructed of 58 mm black square nylon mesh. The pot was divided into upper and lower chambers by a mesh panel that extended midway through the horizontal axis of the pots. A slit in the dividing mesh allowed cod to enter the upper chamber. Zippers were present on the side of both the lower and upper chamber to allow for easy removal of fish and easy re-baiting of the pot. The two entrances of the NOR pot faced each other from opposite directions within the lower chamber, with a single bait bag suspended between them. The modified Norwegian cod pot (NOR-mod pot) was identical in structure to the NOR cod pot described previously (Fig 2B), however we replaced the standard 58 mm mesh surrounding the exterior of the pot, with 100 mm black nylon mesh, which corresponded with the minimum mesh size for commercial cod pots as specified by Fisheries and Oceans Canada (DFO) [31].

The 4-ent pot was a new pot which we designed and constructed (Fig 2C). The 4-ent pot was an intermediate size between the NOR pot and the NL pot (1.5 x 1.5 x 1.2 m), and featured a similar two-chambered, three-ring collapsible structure to the NOR pot. The bottom frame featured two cross beams and was made of 14 mm circular steel (to provide weight on the bottom of the pot), while the two frames above were both made of 14 mm circular aluminum. To provide flotation to the upper rings, we used three 20.3 cm (8 inch) spherical trawl floats with a lifting force of 3.2 kg; two attached to the midway point on opposite sides of the upper aluminum ring and one in the cod-end that floated above the pot similar to the NL and NL mod pot. This allowed the pot to open vertically underwater, with the heavier frame sinking, while the upper frame and floats extended upwards. The pot was divided into two chambers by a mesh separator panel extending at the vertical midway through the horizontal axis of the pot, using 58 mm black nylon netting. A slit in the false bottom mesh allowed cod to enter the upper chamber. The 4-ent pot featured four entrance funnels in the lower chamber made of 58 mm monofilament twine, similar to the NOR pot, and all four entrances face towards a single bait bag suspended in the center of the lower chamber. The exterior of the pot was constructed using two different netting materials, on the bottom and from the lower steel frame to the top aluminum frame we used 100 mm square mesh black polyethylene netting (1.2 to 1.5 mm twine diameter). From the top of the pot to the end of the cod-end which floated above the pot we used 100 mm green polyethylene netting (3 mm twine diameter) hung 50% of stretched mesh opening. We built the 4-ent pot with four entrances based on our previous observation that cod primarily enter pot openings that are aligned with current direction [7], and therefore by having additional entrances, we would increase the likelihood of an entrance being in-line with the current. We embedded zippers on either in the mesh to facilitate removal of any fish snagged in the netting materials, and to allow access to the bait bags so we could re-bait the pot. The 4-ent pots contained a large expandable mesh roof (a cod-end), similar to the NL pot, which extended upward during deployment using a single round trawl float.

### Catch comparison (Year 1–2015)

During our first study, to compare the difference in CPUE between the NL and NOR cod pots, we deployed 15 NL pots and 14 NOR pots along the southern coast of Fogo Island (Fig 1). Our intent was to set each pot every day, so that each pot would be fished for approximately 24 hours per deployment. In practice, due to constraints associated with weather, the needs of our industry partner, and other operational factors, the length of each deployment varied and not all pots could be retrieved each day. We selected deployment sites based on the expertise of our industry partners, selecting sites that they considered to have high densities of cod. We deployed NL and NOR pots in close proximity so that catch rates were comparable across gear types. However, over the course of our study, there were 14 deployments of NL pots that occurred in areas where no NOR pots were simultaneously deployed. Therefore, catch data from these 14 NL pots were excluded from our analysis. At every pot deployment and recovery, we recorded the date, time, latitude, longitude and depth. Differences between latitude, longitude, and depth at deployment and recovery were negligible—i.e. our pots did not move during deployments.

**Fig 3:**
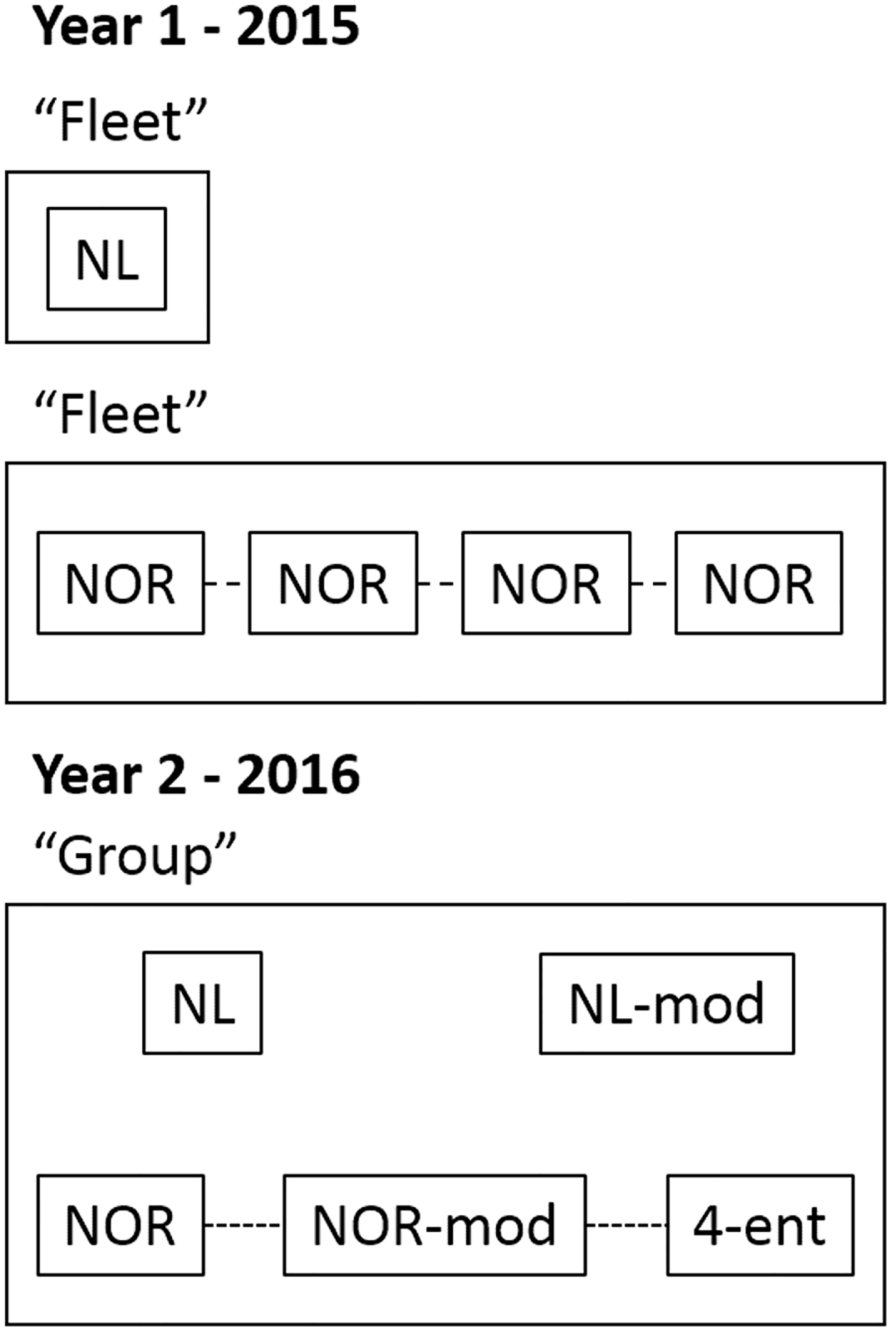
Diagram representing our experimental design for both 2015, and 2016 field studies.

Initially, we fished the NOR pots as one large ‘fleet’ comprised of 14 pots connected by a groundline (Fig 3). This is a viable fishing method for the smaller and lighter Norwegian pots because nesting pots within long strings reduces fuel consumption and handling time (N deployed in 14-pot strings = 28). However, we found that 14 pots were too many to handle on one string, especially when catch numbers were high. Therefore, we switched to fishing three fleets consisting of five, five, and four NOR pots respectively (N deployed in 5-pot strings = 35, and 4-pot strings = 12).

Upon the retrieval of pots, we recorded the total length (TL) of each captured Atlantic cod, and the lengths and species identity of all individuals of non-target fish species caught as bycatch. Fisheries and Oceans Canada (DFO) stipulates that the proportion of landed catch of Atlantic cod below 45 cm in total length (TL) should not exceed 10% in a given fishing area (otherwise the fishing area should be subject to closure; Dave Coffin, Groundfish Resource Manager, DFO, personal communication), therefore we also recorded the number of cod ≤ 45 cm TL in each pot type. We also recorded the number and common name, but not the sizes, of non-target invertebrates caught in each pot. All bycatch species and undersized cod were returned to the water, while the rest of the cod were retained by our industry partner under their commercial fishing license.

Prior to re-deploying pots, we re-baited each pot with a single bait bag containing five frozen squid (*Illex illecebrosus*)–a standard approximate volume used by our industry partners. Squid were used as bait based on the commercial fishing experience of our fishermen partners who have used squid in previous experiments with cod pots [8], as well as previous studies which demonstrated the effectiveness of this bait type [2]. Five frozen squid per deployment was sufficient bait for this experimental design, as previous studies have successfully captured cod using as few as three frozen squid per pot [24].

### Catch comparison (Year 2–2016)

In our second study we compared differences in CPUE across NL, NOR, NL-mod, NOR-mod, and 4-ent pots. In this study (conducted in the same region as our first study), we nested pot deployments within “groups”–i.e. batches of five pots that contained one of each pot type (Fig 3). Our target deployment length was 24 hours, which in some cases was modified by the needs of our industry partner or due to weather. Once again, we selected deployment sites that were likely to produce sufficient catch rates of cod to facilitate comparisons of CPUE across gears.

Because we always had all five pots within a group, fishing sites for all five pots always overlapped, eliminating differences in catch that could occur due to geographical location. In addition, we always retrieved groups in their entirety, ensuring that all pots within a group had nearly identical deployment durations. For every pot deployment and recovery, we recorded the date, time, latitude, longitude, and depth. Deployment depths ranged from 33.5 to 59.1 meters (mean ± 1 SE = 46.0 ± 0.6), and pots rarely moved between deployment and retrieval. Upon the retrieval of pots following a deployment we followed identical procedures as the year 1 study.

### Statistical analysis of catch comparison data

To measure the effect of both pot type and soak duration on CPUE, we used generalized linear mixed-effects models (GLMMs) (Eqs 1 and 2). Our catch data violated many of the assumptions needed for parametric tests due to our catch data not being normally distributed, as well as the fact that NOR pots were nested within fleets during the 2015 field study, and that all five pot types were nested within groups during the 2016 study, and therefore could not be treated as fully independent observations. GLMMs allowed us to measure the effect of pot type on catch rate, while accounting for the non-normal distribution and nested structure of the data [32]. We used mixed effects modeling because our pot deployments were nested within fleets (for the first experiment) and groups (for the second). The distribution of our catch data for both years was best explained by a negative binomial distribution. Residuals met the assumptions for homogeneity, normality, and independence. For our analysis, we treated the standard NL pot as our control treatment, as it was the most used pot by Fogo Island fishers at the time of our study.

For the data collected in 2015, in our initial model, we tested the fixed effects of *pot type* (categorical factor, two levels), *soak duration* (continuous variable), *pot deployment depth* (continuous variable), and tested for an interaction between *pot type* and *soak duration*, with fleet number as a random effect variable (Eq 1). We then conducted stepwise model simplification, dropping non-significant terms one at a time until all terms in the model were statistically significant [33]. This procedure was repeated for data collected in both 2015 and 2016 field studies. Equations are presented below as outlined in Zuur *et al*. (2016) [34]. For data collected in 2016, in our initial model we tested the fixed effects of *pot type* (categorical factor, five levels), *soak duration* (continuous variable), *pot deployment depth* (continuous variable), and tested for an interaction between *pot type* and *soak duration*, with group number as a random effect variable (Eq 2).

$$\begin{array}{l} CatchPerDeployment\;\sim \;NB\left(\mu_{ij}\right)\\ E\left(CatchPerDeployment\right)=\mu_{ij}\\ CatchPerDeployment=\beta_{1}+\beta_{2}\times PotType_{ij}+\beta_{3}\times SoakDuration_{ij}+\beta_{4}\;\times PotType_{ij}\\ \times SoakDuration_{ij}+\beta_{5}\times DeploymentDepth_{ij}+FleetID_{i}\\ FleetID_{i}\;\sim \;N\left(0,\;\sigma^{2}\right) \end{array}$$ (1)

$$\begin{array}{l} CatchPerDeployment\;\sim \;NB\left(\mu_{ij}\right)\\ E\left(CatchPerDeployment\right)=\mu_{ij}\\ CatchPerDeployment=\beta_{1}+\beta_{2}\times PotType_{ij}+\beta_{3}\times SoakDuration_{ij}+\beta_{4}\times PotType_{ij}\\ \times SoakDuration_{ij}+\beta_{5}\times DeploymentDepth_{ij}+GroupID_{i}\\ GroupID_{i}\;\sim \;N\left(0,\;\sigma^{2}\right) \end{array}$$ (2)

We then tested whether pot type affected the mean length of cod that we caught using a general linear model. Mixed effects were not used in this analysis because visual inspection of the data demonstrated no relationship between the sizes of caught fish and group ID, therefore we did not include group ID as a random effect. Body lengths were normally distributed. Therefore, we conducted an ANCOVA on the mean length of cod caught per pot as modeled by a normal distribution. Residuals met the assumptions for homogeneity, normality, and independence. We did all analysis using R statistical software [35].

### Grading receipts (Year 2–2016)

To determine the quality of the cod caught using pots during our field study, we were provided with a small sample of anonymous grading receipts for landings of cod provided to us by the Fogo Island Cooperative Society within the duration of our field study. The grading receipts contained an overall quality score (A, B, or C, in declining order of overall quality), which was based on an assessment of the fillet quality of landed fish. Many factors are considered in these assessments, including parasites, odour, texture, bruising, and colour [36]. The grading receipts we obtained were for the landings of cod caught off the coast of Fogo Island between August 22, 2016, and August 25, 2016 and represent 78 landings of cod from 57 different fishers. Using these grading receipts, we were able to calculate the proportion of catch that was considered grade A, B, and C, for landings of cod, using three different fishing gears (pots, gillnets, and hooks) during the duration of our field study.

## Results

### Catch comparison (Year 1–2015)

We deployed a total of 41 NL pots, and 12 fleets of NOR pots (N = 72 NOR pots). Pot soak times ranged from 19.1 to 95.5 hours (mean ± 1 S.E. = 50.4 ± 2.9) for NOR pots, and 4.4 to 119.7 hours for NL pots (mean ± 1 S.E. = 46.5 ± 4.3). Deployment depths ranged from 25 to 48 m (mean ± 1 SE = 36.7 ± 0.4).

**Fig 4:**
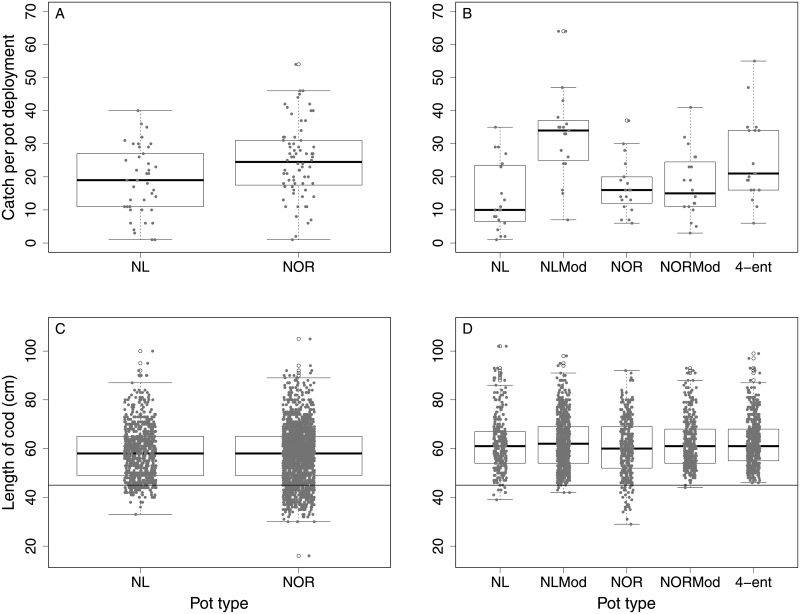
A summary of our catch data and length data collected during both field studies (2015 and 2016). A and C present 2015 data, B and D present 2016 data. Top boxplots (A and B) compare mean catch-per-deployment of Atlantic cod across pot types, where each grey dot represents an individual pot deployment. Bottom boxplots (C and D) compare the total lengths of cod caught between pot types, with each fish indicated as a grey dot. Points below the black line at 45 cm represent fish that were < 45 cm in length.

NOR and NL pots caught means of 24.74 and 18.71 cod per pot deployment respectively (Table 1, Fig 4A). Mean body length of cod did not differ significantly between pot types (p = 0.0792, Fig 4C). Body lengths ranged from 16 to 105 cm (mean ± 1 S.E. = 57.38 ± 0.27) for the NOR pot and 33 to 100 cm (mean ± 1 S.E. = 58.24 ± 0.39) for the NL pot.

**Table 1:** Summary of cod caught per pot type from our 113 deployments.

Pot type	Total caught	Min	Max	Mean CPUE	SE	N
NOR	1781	1	54	24.74	1.33	72
NL	767	1	40	18.71	1.61	41

We found that there was no interaction between pot type and soak duration (β = -0.048, S.E. = 0.090, t = -0.53, *p* = 0.59). Therefore, we dropped the interaction term from the model. In the subsequent models, which did not include the interaction term, we found that effect of both soak duration (β = 0.041, S.E. = 0.044, t = 0.92, *p* = 0.36), and deployment depth (β = -0.032, S.E. = 0.024, t = -1.33, *p* = 0.18) on total catch per pot, were still non-significant. In other words, pots deployed for two or more overnight periods did not catch more cod than pots soaked overnight, and pots placed deeper did not increase or decrease catch. Therefore, for the remainder of our analysis, we dropped soak duration and depth as terms in the model, and treated ‘cod per pot, per deployment’ as our metric of CPUE.

We found that NOR pots caught 32% more cod on average than NL pots (Table 2, Fig 4A), The NL pots caught a total of 67 cod ≤ 45 cm TL (8.7% of catch), and NOR pots caught 278 cod ≤ 45 cm TL (15.6% of catch).

**Table 2:** Estimated regression parameters, standard errors, z-values, and P-values for the negative binomial GLMM presented for catch-per-deployment from our 2015 field study.

	Estimate	Std. error	z value	P-value
Intercept	2.92891	0.08631	33.93	<2e-16
PotTypeNor	0.27936	0.10729	2.60	0.00922

Over the course of this study, we captured six different bycatch species including toad crab (*Hyas* sp.), eelpout (*Lycodes* sp.), Greenland cod (*Gadus ogac*), sculpin (*Myoxocephalus* sp.), green sea urchin (*Strongylocentrotus droebachiensis*), and whelk (*Buccinum undatum*) (Table 3). The most frequently caught bycatch species, for both pot types was toad crab, with 847 caught across all 113 pot deployments.

**Table 3:** Bycatch comparison between the NL and NOR pots for our 2015 field study. Values represent the total number of individuals caught out of 41 NL and 72 NOR deployments. Species with total catch < 10 are excluded from the table.

Common Name (Species)	NOR	NL	Total Catch
Greenland cod (*Gadus ogac*)	0	22	22
Sculpin (*Myoxocephalus* sp.)	8	2	10
Toad crab (*Hyas* sp.)	797	50	847

### Catch comparison (Year 2–2016)

Over the course of the two-week study period (August 20 to Sept 3, 2016), we deployed a total of 125 pots. On August 30, 2016, during our fieldwork, a heavy windstorm affected five groups of deployed pots. Therefore, we removed these five groups and two additional pots (N = 27) from our analysis due to damage that occurred during deployment. Therefore, our final analysis included data from 98 pot deployments, nested within 20 groups, consisting of 20 NL, 20 NOR, 20 NOR-mod, 19 NL-mod, and 19 4-ent pot deployments (Table 4). Soak times for pot deployments ranged from 14.6 to 98.8 hours (mean ± 1 SE = 42.1 ± 2.9).

**Table 4:** Summary of cod caught across all pot deployments for each pot style. N represents the number of pots deployed per pot type.

Pot Type	Total cod caught	Min	Max	Mean CPUE	SE	N
NL	274	1	35	13.7	2.3	20
NOR	339	6	37	17.0	1.8	20
NL-mod	637	7	64	33.5	3.3	19
NOR-mod	351	3	41	17.6	2.2	20
4-ent	476	6	55	25.1	2.9	19

We found that there was no interaction between pot type and soak duration (*p* = 0.056). Therefore, we dropped the interaction term from the model. In the reduced model, which did not include the interaction term, we found that both soak duration (β = 7.65e-04, S.E. = 4.56e-02, t = 0.017, *p* = 0.99), and deployment depth (β = -0.011, S.E. = 0.014, t = -0.77, *p* = 0.44) were non-significant in subsequent reductions. Therefore, for the remainder of our analysis, we dropped duration and deployment depth as terms in the model, and treated cod catch per pot, per deployment as our metric of CPUE.

Statistically, we found that the NL-mod pots caught significantly (145%) more cod on average than the standard NL pot (Table 5, Fig 4B). We found that the 4-ent pot, caught significantly (83%) more cod on average than the NL pot (Table 5, Fig 4B). We also found that both the NOR and NOR-mod pots did not catch significantly more or less cod than the NL pot (Table 5, Fig 4B).

**Table 5:** Estimated regression parameters, standard errors, z-values, and P-values for the negative binomial GLMM presented for catch-per-deployment from our 2016 field study.

	Estimate	Std. error	z value	P-value
Intercept	2.6174	0.127	20.606	< 2e-16
PotType4-ent	0.6036	0.1771	3.407	0.000656
PotTypeNLMod	0.8949	0.1756	5.096	3.48E-07
PotTypeNOR	0.2129	0.1777	1.198	0.230875
PotTypeNORMod	0.2477	0.1774	1.396	0.162679

Mean body length of cod did not differ significantly between pot types in comparison to the standard NL pot for all five pot types (Fig 4D), NL-mod (p = 0.390), NOR (p = 0.101), NOR-mod (p = 0.697), and 4-ent pot (p = 0.503). Body lengths of cod are presented in Fig 4D.

The proportion of catch below 45 cm was 2.55% for the NL pot, 0.63% for the NL-mod pot, 7.37% for the NOR pot, 0.39% for the NOR-mod pot, and 0.00% for the 4-ent pot. The standard NOR pot had the greatest proportion of catch < 45 cm (Fig 4D), whereas the 4-ent pot had no catch below 45 cm. We reduced the undersized catch (< 45 cm) by 95% by switching to a bigger mesh size in NOR pots.

Over the course of the second field study, we captured seven different bycatch species including toad crab, eelpout, Greenland cod, sculpin, urchin, whelk, and harbor seal (*Phoca vitulina*) (Table 6). The most frequently caught bycatch species for all five pot types was toad crab. A single seal was caught in one deployment within an NL-mod pot, which had an unusually long deployment (4 days) due to poor weather inhibiting our ability to retrieve the pot.

**Table 6:** Bycatch comparison between all five pot types for our 2016 field study. Values represent the total number of individuals caught out of 20 NL, 20 NOR, 19 NL-mod, 20 NOR-mod, and 19 4-ent pot deployments (98 pot deployments total). Species with a total catch < 10 are excluded from the table.

Common Name (Species)	NL	NL-mod	NOR	NOR-mod	4-ent	Total Catch
Toad crab (*Hyas* sp.)	54	401	512	589	810	2366
Greenland cod (*Gadus ogac*)	1	8	3	6	3	21
Sculpin (*Myoxocephalus sp*.)	15	8	13	14	17	67

### Grading sheets

**Fig 5:**
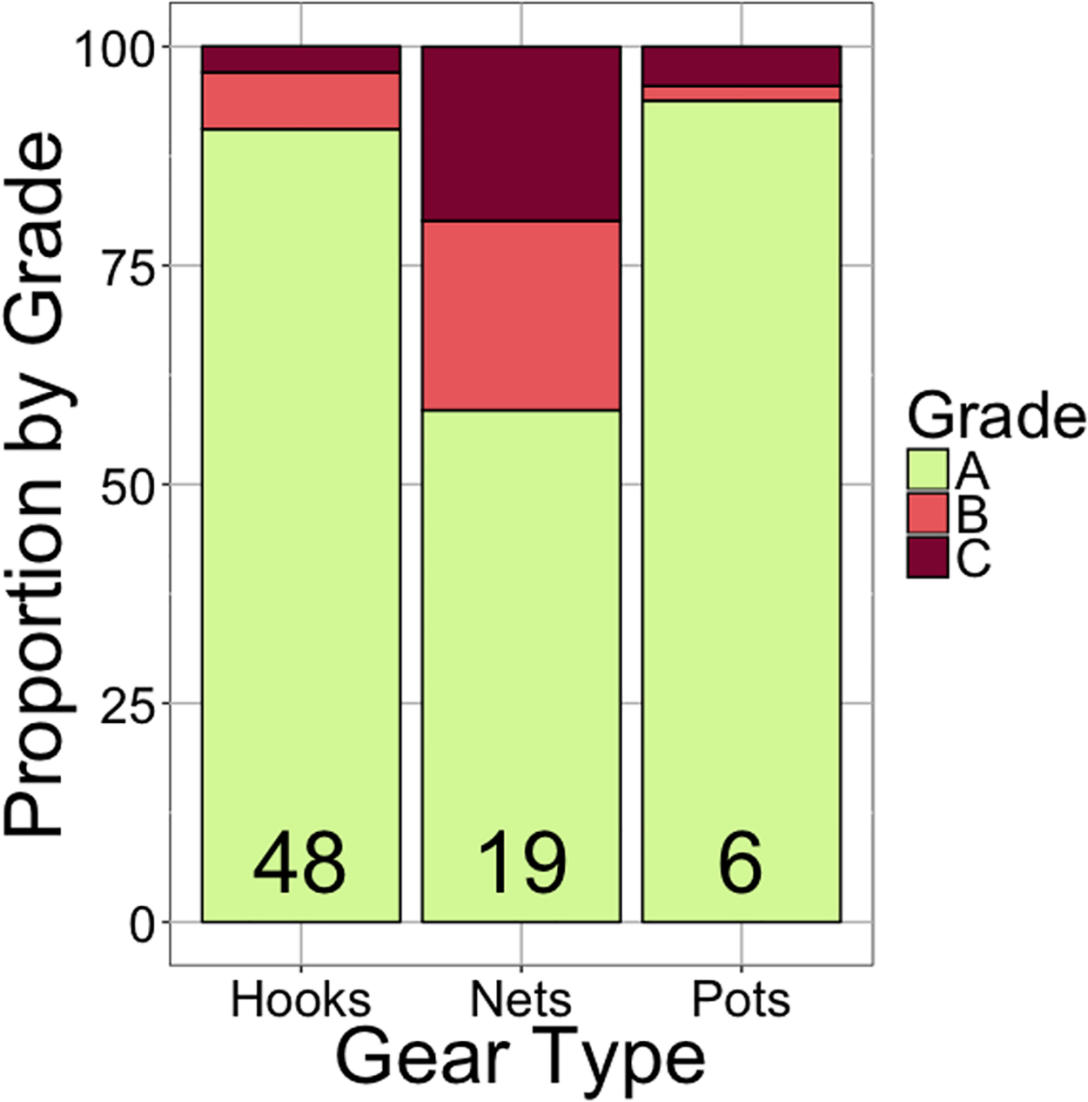
The proportion of cod landings considered grade A, B, or C quality for hooks, gillnets, and pots during our field study. Numerical values represent the sample size, which was the number of grading receipts for the respective fishing gear type within our study period.

A total of 94% of the landings from cod pots were ranked as grade A, while hooks produced 91% grade A landings. Gillnets produced only 58% grade A catch (Fig 5).

## Discussion

In our 2015 study, we found that the NOR pot clearly outperformed the NL pot. This provided the first evidence that lightweight pots could be useful in the NL cod fishery. The difference in CPUE may have been a result of design features of the NL pot such as the metal retention triggers, which, as we have previously reported, appear observed to deter the entry of cod [7]. The mean body length of cod did not differ significantly between the NOR and NL pots, but since the NOR pots caught more fish overall, the pots accumulated a larger absolute number of undersized (< 45 cm) cod than the NL pots. This was our primary motivation for increasing the mesh size for the NOR pot in our second field study in 2016, through the NOR-mod pot. Regardless, our conclusion from year 1 was that NOR pots were clearly more effective at catching cod than the NL pots.

In the second year, we found that the modified NL pot was the best performer—even outperforming the 4-ent pot. Our expectation was that the presence of entrances on all sides of the 4-ent pot would result in an entrance always facing the down-current direction, thereby making it easier for cod to enter pots (see: [7]). While that likely worked, the addition of extra openings also facilitates exit, and it is possible that the exit rate increased more than the entrance rate. In another field study in the Barents Sea, researchers found that floated pots—which could reorient themselves based on current direction—with two entrances caught 82% fewer cod per deployment than one-entrance pots [37], thus demonstrating the importance of exit rate as a determinant of final catch. A second possible explanation for the higher CPUE of the NL-mod pot is that cod tend to swim upwards inside pots [7]. Each entrance had associated twine that crisscrossed the interior of the pot, and having four entrances potentially created barriers to the ability of trapped cod to swim upward and into the cod-end.

The modifications made to the NL pot had a major impact on catch rates, particularly versus the unmodified NL pots. By removing the metal retention triggers, changing the entrance funnel design, and adding the mesh separator, we were able to substantially increase the catch of the NL-mod pot relative to the standard NL pot. The key modifications to the NL pots were inspired by features from the NOR pots. The design factor that we believe was most responsible for the improved catch rates was the inclusion of the mesh separator panel as the primary retention mechanism for trapped cod, as opposed to metal triggers on the entrances, which had been observed to deter the entry of cod [7].

Overall, we found that all five pot types were effective at catching substantial quantities of Atlantic cod off the coast of Fogo Island during the commercial fishing season. However, NL-mod and 4-ent pots produced the highest CPUE, and that increasing the mesh size in NOR pots essentially eliminated undersized catch. There are two main implications of these findings. First, there is substantial opportunity for variation among potentially effective pot designs, in terms of size, shape, and dimension. This suggests that fishers can have a degree of flexibility in building and deploying pots that work well for the deck configuration of their fishing vessels. Second, to maximize catch rate while minimizing impact on habitat and non-target species, designs should incorporate several features. First, pots should use clear nylon monofilament entrance funnels, with bait bags suspended on the interior end in line with the entrances. Second, pots should be built with 100 mm mesh on the exterior of the pots to reduce undersized bycatch. Third, a mesh separator panel that divides the pot into two chambers should be included at the midway point of the interior of the pot, with a slit that allows the movement of fish between chambers.

In other studies that employed field tests of cod pots, researchers have noted lower CPUE for NOR pots than we experienced here. Specifically, [38] caught only 231 cod across 377 deployments, resulting in a CPUE of only 0.61 cod per pot deployment, in contrast to our CPUE of 24.7 and 17.0 cod per NOR pot deployment for our 2015 and 2016 field studies respectively. Likewise, floated NOR pots with one and two entrances caught 4.11 and 2.70 cod per deployment, respectively [37]–with both values being lower than our CPUE for two-entrance NOR pots in both years. Clearly, these studies were conducted at different times, in different ecosystems, and on different populations of Atlantic cod. Nevertheless, these differences indicate that fishers may expect variable catch rates across ecosystems, and it is possible that pot designs may need to be customized for the system in which they are used. Therefore, further research into the optimal design of cod pots for different populations should be an ongoing effort.

It remains unclear what the optimal soak duration is for cod pots in NL. In our study, there was no relationship between soak duration and CPUE– 24 h was just as effective as longer deployments. However, longer soak durations have been associated with higher catch rates in other fisheries [28]. Nevertheless, an advantage of pots over other fishing gears is that fish trapped within pots do not die until retrieval. This means that if fishers are unable to retrieve pots (e.g. due to bad weather) they will not lose quality of catch due to *in situ* decomposition. This provides fishers the ability to catch quotas quickly (with short soak times and daily retrieval of gear), or to stretch out the fishery over a longer time period (with long soak times and sporadic retrieval of gear). Either strategy could potentially be used without compromising fish quality.

The amount of pre-slaughter stress experienced by fish has been linked to quality [39], and stress varies depending on the fishing gear [40,41]. To ensure high quality catches, capture techniques that minimize stress are desirable. Recent findings have suggested that pot-caught cod suffered from less stress during capture and handling than longlines [41]. In addition, in a previous study we found most cod within pots showed no obvious visual signs of stress [7], which is supported by a study that observed captured cod resting within pots [28]. The data provided to us by the processor suggested, at least within the limited sample size examined, that professional fish graders reported higher quality among pot-caught cod than cod caught using gillnets. This is unsurprising, because previous research has also demonstrated that cod caught using gillnets consistently received the lowest prices compared to other gears [42]. Stress in fish has been correlated with reduced market quality [39] and so the higher quality grades in pot-caught-cod during our study period, suggests the possibility that these fish were not subject to high stress during the capture process.

Over the course of our two studies, we caught a low diversity of non-target species (N = 7), with our only major bycatch by count being toad crab. In contrast, a similar study between NL and NOR cod pots in Massachusetts caught an increased diversity of bycatch with 15 different species caught during their study period [38]. Toad crab is currently classified as Least Concern by the International Union for the Conservation of Nature [43], and local populations of toad crab are likely robust to the effects of capture using pots, as seen in other species of crab e.g. [44]. Nevertheless, it is good practice to minimize impacts on non-target species, even if they are least concern. One option would be to focus further gear modification efforts to reduce crab bycatch, such as using floated pots which were observed to reduce the bycatch of king crabs in the Barents Sea [24], while another would be to acknowledge this bycatch within the conditions of fishing licenses, and manage as a multi-species fishery [45,46].

While the environmental case for a shift from gillnets to less impactful gears is clear, environmental benefits alone are rarely sufficient to motivate change within an industry. When adopting alternative fishing gears, achieving greater, or at least comparable catches, with similar input effort is an important factor for fishers to consider abandoning traditional gears [29]. There are three non-environmental reasons that a shift to using pots would make sense for a fishing operation. First, the presence of zippers and a cod-end in the design of pots means that there is less labor required to remove the catch from pots, in contrast to gillnets which require greater effort [47]. Second, because the fish are freely swimming within the pots until recovery, fishermen have the flexibility to retrieve their gear at their own convenience, avoiding the risk of hauling their gear during inclement weather. Thirdly, if a market could be established for higher quality cod, and fishermen rewarded with a greater price-per-kilogram for high quality fish caught using pots e.g. [48,49], then the financial gains a fisher could make from pots would be substantial when compared to gillnets.

Despite signs of limited recovery [16], the northern cod stock remains depressed [15]. It is critical that any decision made about exploitation of northern cod be precautionary in nature, and be considered in an ecosystem context [50]. Empowering managers and fishers to use gears, such as pots, that produce reduced impacts on ecosystems while meeting the needs of industry is a key aspect to promoting sustainable management of cod. These results show that pots are effective at catching cod while minimizing catch of non-target species, and that modifications to gear can increase catch efficiency while decreasing bycatch. Different pot types can produce substantially different catch rates. The different catch rates we observed across pot designs, fishing at the same time of year in the same locations, demonstrates that innovation within this class of fishing gear can substantially improve its usability as a tool for industry.

## Supporting information

S1 File Cod pot field data 2015.xlsx. Total catch and length data collected for marine species caught using experimental cod pots during the August and September 2015 field season.
(XLSX)

S2 File Cod pot field data 2016.xlsx. Total catch and length data collected for marine species caught using experimental cod pots during the August and September 2016 field season.
(XLSX)
